# Deprivation and poor psychosocial support are key determinants of late antenatal presentation and poor fetal outcomes-a combined retrospective and prospective study

**DOI:** 10.1186/s12884-015-0753-3

**Published:** 2015-11-25

**Authors:** Habiba Kapaya, Erin Mercer, Francesca Boffey, Georgina Jones, Caroline Mitchell, Dilly Anumba

**Affiliations:** Department of Human Metabolism, Academic Unit of Reproductive & Developmental Medicine, 4th Floor Jessop Wing, Tree Root Walk, Sheffield, S10 2SF UK; Health Economics & Decision Science, School of Health & Related Research, University of Sheffield, Regent Court, 30 Regent Street, Sheffield, S1 4DA UK; Academic Unit of Primary Medical Care, The Medical School, University of Sheffield, Sheffield, UK

**Keywords:** Pregnancy, Antenatal care, Access, Late booking, Social exclusion, Deprivation

## Abstract

**Background:**

Published guidelines emphasise the need for early antenatal care to promote maternal and neonatal health. Inadequate engagement with antenatal care is associated with adverse pregnancy outcomes including maternal death. The factors that influence the uptake and utilisation of maternity care services are poorly understood. We retrospectively explore a large maternity database of births in a large referral UK hospital to capture the socio-demographic factors that influence late pregnancy booking, and then prospectively compare the stress and social support status of consenting early and late-booking women.

**Methods:**

Retrospective socio-demographic and clinical outcome data on 59,487 women were collected from the maternity database record of births between 2002 and 2010 at the Jessop Wing Hospital, Sheffield UK. In a follow-on prospective survey between October 2012 and May 2013 a convenience cohort of early and late bookers for antenatal care were then studied using validated scales for fetomaternal attachment, stress and anxiety, and social support.

**Results:**

In our retrospective study, pregnancy during the teenage years, higher parity, non-white ethnic background, unemployment and smoking were significantly associated with late access to antenatal services and poor fetal outcomes (*P* < 0.001). However, late booking per se did not predict adverse fetal outcomes, when socio-demographic factors were accounted for. A high index of multiple deprivation (IMD) score remained independently associated with late booking when confounding factors such as ethnicity and employment status were controlled for in the model (*P* = 0.03). Our prospective data demonstrated that women who book late were more likely to be unmarried (OR: 3.571, 95 % CI: 1.464–8.196, *p* = 0.005), of high parity (OR: 1.759, 95 % CI: 1.154–2.684, *P* = 0.009), and have lower social support than early bookers (*P* = 0.047).

**Conclusions:**

Of the many complex sociocultural factors that influence the timing of maternal engagement with antenatal care, multiple deprivation and poor social support remain key factors. Improving access to prenatal care requires in-depth exploration of the relationship between maternal psychosocial health indices, social support mechanisms and engagement with antenatal care. Findings from these studies should inform interventions aimed at improving access to care.

**Electronic supplementary material:**

The online version of this article (doi:10.1186/s12884-015-0753-3) contains supplementary material, which is available to authorized users.

## Background

Antenatal care is widely acknowledged as contributing to improved pregnancy outcomes, with delayed access (“late booking”) linked to increased maternal, fetal and infant mortality and morbidity [1, 2]. Over the last decade the confidential enquiries into maternal deaths (CEMD) in the United Kingdom (UK) have identified “late booking” as a significant risk factor for poor pregnancy outcomes [3, 4]. The UK National Institute of Health and Care Excellence (NICE) and the UK Department of Health have published guidelines which emphasise the need for early attendance for antenatal care, with the suggestion that the first visit should happen before 12 weeks gestation [5]. The booking visit enables a full risk assessment surrounding the health of the mother and the baby, including a psychosocial history. Early booking enables the initiation of prenatal fetal and maternal screening and the provision of health advice on life-style issues during pregnancy, such as healthy nutrition, exercise, alcohol and smoking cessation amongst others. However, many women do not present early for antenatal booking, with a few women first attending hospital when they go into labour [6].

Observational studies have suggested that ‘late bookers’ for antenatal care are typically from socially marginalised groups; non-white ethnicity in particular. Young age, low income and educational level, lack of support and substance misuse have also been found to be common characteristics in this group of women [1, 7–10]. In addition to ethnicity and social factors, individual attendance for antenatal care may be influenced by the woman’s own feelings about herself (self-esteem), her baby (attachment), planning of pregnancy and her acceptance of the reality of the pregnancy (psychological status) [11–13]. These dimensions have been inadequately explored in relation to antenatal attendance.

A meta-analysis of UK studies [6] assessing attendance for antenatal care has highlighted a dearth of good quality research identifying the factors that contribute to poor antenatal attendance according to any measures of social class, deprivation, exclusion or ethnicity. The reported studies were noted to be of “poor methodological quality”, and did not control for potential confounding factors such as age, parity and clinical risk factors. New studies are necessary that investigate the impact of social exclusion factors on access to maternity services and other clinical and psychosocial outcomes.

To elucidate the influence of late access to antenatal care on pregnancy outcomes, we initially undertook a retrospective observational database study of late antenatal bookers at the Jessop Wing (JW) Hospital in Sheffield, UK from January 2001 till December 2010. We then sought to explore through a pilot study, the inter-relationships between maternal fetal attachment, social support, anxiety, self-esteem and general health attitudes and the timing of initiation of antenatal care. Our retrospective and prospective observations are detailed in this study.

## Methods

Retrospective socio-demographic and clinical outcome data on 59,487 women were collected from the maternity database record of births between 2002 and 2010 at the Jessop Wing Hospital, Sheffield UK. In a follow-on prospective questionnaire-based survey study between October 2012 and May 2013 a cohort of early and late bookers who attended for antenatal care were then opportunistically recruited and studied using validated scales for fetomaternal attachment, stress, anxiety and social support.

This study was approved by the NRES ethics committee for Yorkshire and Humber with REC number 11/YH0372. Permission to access the medical records of the Jessop Wing Maternity Hospital used in the retrospective part of the study was granted by the Research and Development Department of the Sheffield Teaching Hospitals Trust.

### Study design

#### Retrospective service evaluation

We collected retrospective data from the records of 59,487 consecutive singleton births at the JW Maternity Hospital, Sheffield, UK between January 2002–December 2010. The data included maternal demographic features as well as clinical and neonatal outcomes. We then compared women who presented for pregnancy care early before 14 weeks gestation (the early bookers,-EBs) to those who presented after 20 weeks gestation (the late bookers,-LBs). We employed regional ordinance maps showing the Index of Multiple Deprivation (IMD) scores across South Yorkshire to determine IMD scores for all women during the period, categorised by whether they booked early or late for antenatal care. The IMD score is a measure of deprivation in small geographical areas of the UK [14]. The scores for each area are based upon various factors; income, health and disability, employment, barriers to housing and other services, crime, living environment and education, skills and training. Therefore, IMD is very useful in research into populations because it encompasses many factors by which an individual may be subject to adversity. Whilst a high score is indicative of high deprivation for the area, low IMD scores are associated with better socioeconomic statuses and higher education [14]. For each mother IMD score was calculated according to the Lower Super Output Area (LSOA) in which their postcode of residence fell. On average, LSOAs have a population of 1500 women and are generated using data from the 2001 census [15], list of which was obtained from the UKBORDERS website [16].

#### Prospective assessment of indices of social support and fetomaternal attachment

A questionnaire-based survey methodology was used for this study. Women who attended for antenatal booking at the JW Hospital, between October 2012 and May 2013 were approached and informed of the study via an advert leaflet for recruitment with triangulation of data collection from the antenatal record. Interested participants were then recruited following discussion with a member of the research staff. Women gave written informed consent to participate in the study as well as to gain access to medical records. Our study group comprised a cohort of mothers booking late, defined for the purpose of this study as presenting for the first time for antenatal care in the hospital >14 weeks gestation, and a control group which included women who attended their booking appointment before 14 weeks gestation.

Two hundred forty-one women consented to this aspect of study and received questionnaires. We employed a survey instrument to capture validated measures of prenatal attachment, social support, psychological health, self-esteem and general health. The following widely accepted scales were employed: the Maternal Antenatal attachment Scale (MAAS) [17], the Maternity Social Support scale (MSSS) [18], the State Trait Anxiety Inventory scale (STAI) [19, 20], the Rosenberg Self-Esteem scale (RSES) [21, 22] and the Short Form Health Survey version (SF-12) [23] respectively. The MAAS is a 19-item instrument that measures prenatal attachment and take no more than 5–10 min to administer. All of the items are scored on a 5-point Likert scale; ranging from “very weak” to “very strong”. The MSSS is quantified by using 6-item questionnaire and takes no more than 5 min to administer. Each statement has a 5-point Likert scale ranging from “always” to “never”. Factors that are associated with postnatal depression such as conflict with partner, controlled by spouse/partner, feeling unloved, low friendship and family support are quantified. The STAI measures the emotional reactions in terms of anxiety at a particular moment or period of time and take no more than 5 min. Statements are on a 4-point Likert scale of increasing intensity, from “not at all” to “very often”. The RSES is a 10-item questionnaire and takes no more than 3–5 min to complete. Each statement has a 4-point Likert scale ranging from “strongly agree” to “strongly disagree”. The SF-12 is a shorter version of SF-36; captures both physical and mental health status and takes around 2 min for completion. The physical health section looks at physical functioning, body pain and general health, whereas the mental health looks at vitality, social functioning, emotional role and mental health. The scores range from 0 to 100, with 100 indicating the highest level of health. A front sheet for the validated questionnaires was developed by the team and comprised a socio-demographic questionnaire with closed responses.

Descriptive statistics were employed to summarise quantitative data for both studies. Categorical outcomes were compared using Chi-squared test, whereas Independent-samples t tests or Mann Whitney tests were employed for the continuous outcome measures. To explore the relationship between socio-demographic data, psychosocial factors, fetal outcome and booking status, binary logistic regressions were performed. Adjustment for potential confounding variables was carried out using multivariate analysis, whereby the association of early and late booking with outcomes and psychosocial support scales was examined while accounting for the demographic variables. These analyses were done using SPSS version 19 and the level of significance was set at *P* < 0.05.

## Results

### Retrospective study

Overall, data for 59,487 women were included in the study. 29, 698 women (49.9 %) had their antenatal appointment before 14 weeks and were classified as early bookers (EBs), whereas 4686 women (7.9 %) had their antenatal booking appointment after 20 weeks gestation and so were considered as late bookers (LBs). Data regarding the booking appointment was incomplete for 5096 women (8.6 %).

The socio-demographic characteristics of the women are summarised in Table 1. The mean age of EBs and LBs were similar (28.7 ± 6 in EBs and 27.5 ± 6.6 in LBs), but more women in the late booking group were teenagers compared to EBs (12.4 % vs. 6.9 %, *P* < 0.001). On the other hand more women aged above 35 years were EBs than the LBs (18.0 % vs. 16.4 %, *P* = 0.01). The majority of women in the two groups were white, (83.7 % EBs, 62.6 % LBs), but the proportion of women from non-white backgrounds was much higher for LBs (37.4 %), compared to the EBs (16.3 %, *P* < 0.001).

**Table 1:** Demographic characteristics of retrospective study

Variables	Early bookers *n* = 29,698	Late bookers *n* = 4686	Significance
Age (years): mean (range;SD)	28.7 (14–52; 6)	27.5 (13–32; 6.6)	*P* = 0.61
Teenage pregnancy = Yes : %(*n*)	6.9 (2031)	12.4 (579)	*P* < 0.001
Maternal age > 35 years = Yes: %(*n*)	18.0 (5321)	16.4 (769)	*P* = 0.011
Parity: mean	0.85	1.14	*P* < 0.001
White ethnicity = No: %(*n*)	16.3 (4750)	37.4 % (1668)	*P* < 0.001
Maternal employment = No: %(*n*)	23.9 (4263)	52 (1254)	*P* < 0.001
Paternal employment = No: %(*n*)	4.8 (753)	12.4 (227)	*P* < 0.001
IMD score % (*n*)	5.9 (1068)	10.4 (1875)	=0.031
Smoking status = Yes: %(*n*)	14.7 (4348)	18.8 (873)	*P* < 0.001
Body mass index (kg/m^2^): mean (range, SD)	25.6 (17.5–52; 4.7)	26.3 (17.6–54; 4.6)	*P* = 0.357

A greater proportion of women and their partners were employed in the early booking group (54.0 %, 88.9 %) compared to the late booking group (32.1 %, 76.1 %, *P* < 0.001). Women who smoked during pregnancy were higher in the late booking group (18.8 % vs 14.7 % in EBs, *P* < 0.001). A large percentage of women in late booking group belonged to highly deprived areas and scored high on IMD (10.4 %) vs 5.9 % in the low IMD score category, *P* = 0.03).

Binary logistic regression analysis was performed to assess the correlation of socio-demographic factors and pregnancy outcome on booking status. This is illustrated in Table 2. The odds of late antenatal booking was twice as high for teenage parturients compared to older women (OR: 1.919, 95 % CI: 1.740 to 2.117, *P* < 0.001). Similarly, women from non-white ethnic background were thrice at higher odds of booking late compared to white ethnic women (OR: 3.07, 95 % CI: 2.9 to 3.28, *P* < 0.001). Furthermore, the odds of an unemployed mother and her partner being a late booker was 3.12 and 2.48 times higher than among EBs (OR: 3.74, 95 % CI: 3.43 to 4.10, *P* < 0.001) and (OR: 2.92, 95 % CI: 2.49 to 3.42, *P* < 0.001) respectively. IMD was found to be a significant independent predictor for late booking (OR: 1.092, 95 % CI: 1.008 to 1.182, *P* = 0.031) when the outcome was adjusted for confounding factors such as ethnicity, smoking status, maternal age, parity and couple employment status.

**Table 2:** Logistic regression of socio-demographics in predicting booking appointments and pregnancy outcome. (Reference group is late booking)

Characteristics	Early bookers *n* = 29,698	Late bookers *n* = 4686	Odds ratio [95 % confidence interval]	Significance
Age <19 years	*n* = 1200 (4.0 %)	*n* = 362 (7.7 %)	1.919 [1.740 to 2.117]	*P* < 0.001
Non-white ethnic background	*n* = 4750 (16.0 %)	*n* = 1668 (35.6 %)	3.07 [2.9 to 3.28]	*P* < 0.001
IMD score	*n* = 9257 (31.1 %)	*n* = 1875 (40.0 %)	1.092 [1.008–1.182]	*P* < 0.001
Maternal un-employment	*n* = 4263 (14.4 %)	*n* = 1254 (26.8 %)	3.74 [3.43 to 4.10],	*P* < 0.001
Partner un-employment	*n* = 753 (2.5 %)	*n* = 227 (4.8 %)	2.92 [2.49 to 3.42]	*P* < 0.001
Smoking status	*n* = 4348 (14.6 %)	*n* = 873 (18.6 %)	1.34 [1.24–1.46]	*P* < 0.001
Stillbirth	*n* = 155 (0.5 %)	*n* = 52 (1.1 %)	2.21 [1.53 to 2.87]	*P* < 0.001
Low birth weight	*n* = 2133 (7.2 %)	*n* = 491 (10.5 %)	1.52 [1.37 to 1.61]	*P* < 0.001
Premature delivery	*n* = 2103 (7.1 %)	*n* = 484 (10.3 %)	1.51 [1.36–1.68]	*P* < 0.001
Admission to SCBU	*n* = 1815 (6.1 %)	*n* = 482 (10.3 %)	1.7 [1.53 to 1.89]	*P* < 0.001

Our data demonstrated higher incidence of stillbirth, (OR: 2.21, 95 % CI: 1.53 to 2.87, *P* < 0.001), low birth weight, (OR: 1.52, 95 % CI: 1.37 to 1.61, *P* < 0.001), premature delivery, (OR: 1.51, 95 % CI: 1.36 to 1.68, *P* < 0.001) and admission to special care baby unit (SCBU) (OR: 1.7, 95 % CI: 1.53 to 1.89, *P* < 0.001) in LBs.

To explore whether adverse fetal outcomes were due to their late booking status or whether socio economic factors were responsible for this difference, further analyses were performed by combining demographic variables and booking status together in the model. Our results showed that none of the adverse fetal outcomes was predicted from the booking status. However, high IMD scores, increased maternal age, and non-white ethnic background increased the risk of having a baby with a birth weight less than 2.5 kg by 1.7 (OR: 1.664, 95 % CI: 1.307 to 2.119, *P* < 0.001), 1.03 (OR: 1.029, 95 % CI: 1.008 to 1.049, o = 0.006) and 1.7 times (OR: 1.663, 95 % CI: 1.272 to 2.174, *P* < 0.001) respectively. On the other hand, every unit rise in parity and non-smoking status reduced the risk of low birth weight by 80 % (OR: 0.878, 95 % CI: 0.789 to 0.977, *P* = 0.017) and 50 % (OR: 0.414, 95 % CI: 0.317 to 0.541, *P* < 0.001) respectively. Smokers were 59 % (OR: 0.627, 95 % CI: 0.475 to 0.828, *P* < 0.001) and women residing in the high IMD score locations of Sheffield were 34 % (OR: 1.338, 95 % CI: 1.055 to 1.698, *P* = 0.017) more likely to have a new-born before 37 weeks gestation. Being a non-smoker almost halved the risk of having a baby admitted to SCBU (OR: 0.546, 95 % CI: 0.407 to 0.733, *P* < 0.001). None of the socio-demographics predicted stillbirth.

### Prospective study

Two hundred and ninety four women were approached to take part in the study. From those approached, 241 (82 %) women consented for participation and were given questionnaires to complete. Of these 169 (70.1 %) booked before 14 weeks and were considered as early bookers (EBs) and 72 (29.8 %) booked after 14 weeks gestation and were classified as late bookers (LBs).

**Fig. 1:**
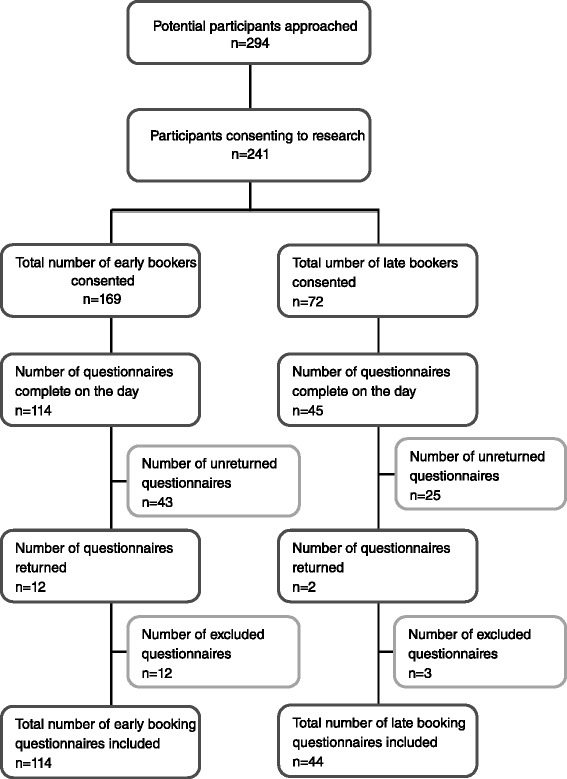
Flow diagram showing recruitment (Prospective study)

Figure 1 represents the recruitment process. The overall final response rate of completed questionnaires was 158/241 = 65.6 %. A total of 114/169 (67.5 %) women completed questionnaires in the early booking group and 44/72 (61.1 %) women in the late booking group.

The demographic characteristics for participants in the two groups are illustrated in Table 3. No participants were aged below 16 years. All demographic variables were broadly comparable between the groups except for parity and marital status. Late booking women were more likely to be unmarried and of high parity than EBs (OR: 3.571, 95 % CI: 1.464–8.196, *p* = 0.005) and (OR: 1.759, 95 % CI: 1.154–2.684, *p* = 0.009) respectively.

**Table 3:** Demographic characteristics of prospective study

Variables		Early booking *n* = 114	Late booking *n* = 44	Significance
Age (years): mean (range;SD)		29.75 (17.62–40.20;5.33)	29.89 (18.40–43.88;6.42)	*P* = 0.893
Teenage pregnancy = Yes : % (*n*)		4.4 (5)	4.5 (2)	*P* = 0.965
Maternal age > 35 years = Yes: % (*n*)		19.3 (22)	25.0 (11)	*P* = 0.429
Parity: median (range)		0 (0–4)	1 (0–6)	*P* = 0.046
Multiparous: % (*n*)		49.1 (56)	59.1 (26)	*P* = 0.261
Married/Partnership = No: % (*n*)		43.9 (50)	65.9 (29)	*P* = 0.013
White ethnicity = No: % (*n*)		27.2 (31)	25.0 (11)	*P* = 0.780
Employment = No: % (*n*)		21.6 (24)	31.8 (14)	*P* = 0.183
Further education: % (n)	No qualifications	2.7 (3)	4.7 (2)	
	Up to NVQ level 2	28.3 (32)	32.6 (14)	
	Up to NVQ level 3	15.9 (18)	25.6 (11)	*P* = 0.440
	Up to professional qualifications	46.0 (52)	32.6 (14)	
	Foreign qualifications	7.1 (8)	4.7 % (2)	
Smoking status = Yes: %(*n*)		8.8 (10)	15.9 (7)	*P* = 0.201
Body mass index (kg/m^2^): mean (range, SD)		25.12 (17.54–42.24;5.21)	22.96 (18.59–30.47;3.38)	*P* = 0.082

In response to questions on the fetal attachment scale (MAAS), there was no significant difference in the scores for any of the parameters on the scale between early and late bookers (refer to Additional file 1: Appendix 1) except for the following question:

### “Over the past 2 weeks when I have spoken about, or thought about the baby inside me I got emotional feelings (scores on a 5-point Likert scale ranging from “very weak” to “very strong”)

Early bookers had stronger emotions when speaking or thinking about their baby and their mean score for this question was 3.89 compared to late bookers (mean of 3.50; *P* = 0.008). When maternal social support scale (MSSS) and timing of booking visit was compared, we found higher mean scores for questions on support from family (4.75), friends (4.49) and husband/partner (4.77) in early bookers compared to late bookers (4.45, 4.43, 4.43). However, results did not attain statistical significance for any of the above (see Additional file 2: Appendix 2). On the other hand, when response on conflict with husband/partner was compared between the two groups, we found a significantly larger range of responses from late bookers (0–5) compared to early bookers (2–5), *P* = 0.042. Comparing self-esteem and anxiety data, using RSES (Additional file 3: Appendix 3) and STAI scale (Additional file 4: Appendix 4) both demonstrated almost similar results between the two groups.

A small difference was observed for the question ***“I feel strained***” on the anxiety scale, with early bookers having a high mean score (1.65) compared to late bookers (1.39). However median value and response range was similar between the two groups (1, 1–4), suggesting an insignificant statistical difference (*P* = 0.067). Assessment of general health employing the physical and mental health scale revealed higher mean scores for late compared to early bookers (see Additional file 5: Appendix 5). Late bookers scored 47.22 for physical health status and 50.62 for mental status, compared to 43.96 and 49.04 for EBs.

To explore relationship between socio-demographics and scores on maternal attachment, social support, anxiety status, self-esteem and general health, binary logistic regression analysis was performed. For early bookers, being unemployed increased the risk of having low maternal fetal attachment (OR: 3.499, 95 % CI 1.158–10.566, *P* = 0.026), whereas for late bookers being multiparous increased the risk of having low maternal fetal attachment (OR: 10.29, 95 % CI: 1.586–66.756, *P* = 0.015). However, none of the socio-demographic variables predicted social support level in either early or late bookers.

Unemployment and being unmarried were significant predictors for anxiety and low self-esteem in early bookers, but not in late-bookers. Being unemployed increased the risk of having anxiety (OR 6.581, 95 % CI: 1.642–26.383, *P* = 0.008) and being unmarried increased the risk of having low self-esteem (OR 5.689, 95 % CI: 1.092–26.622, *P* = 0.039).

On assessing relationship in scores of different scales between the two groups, we found a positive correlation between maternal social support and fetal-attachment (*r*^2^ = 0.090), self-esteem (*r*^2^ = 0.277) and perceived mental health (*r*^2^ = 0.188) and a negative correlation with anxiety scores (*r*^2^ = 0.279) in both groups (*P* < 0.001).

We collected socio-demographic data from 83/241 women who consented to participate but were excluded from the study as they either did not complete or return questionnaires. Binary logistic regression was performed to determine if/whether any of the variables predicted non-recruitment. Our data showed that teenage pregnancy and non-white ethnicity increased the odds of being unrecruited by 13 times (OR: 13.027, 95 % CI: 1.132–150.015, *p* = 0.040) and 17 times (OR: 17.075, 95 % CI: 2.980–97.832, *P* = 0.001) respectively.

## Discussion

This is the first paper to report retrospective and prospective data highlighting the association between psychosocial factors of pregnant women and the timing of their booking appointment. Our retrospective data showed that teenage pregnancies, increased parity, non-white ethnic background, unemployment, smoking status and high IMD score were significantly associated with delayed access to antenatal services and poor fetal outcomes. Furthermore, the data has shown that high IMD score is independently associated with late booking when other confounding factors such as ethnicity, employment status etc. were controlled in the model.

Findings from our study confirm the conclusions of other studies regarding the importance of factors of social exclusion on poor fetal outcomes and reconfirm the findings of numerous studies that there exists a subset of women who face difficulties in accessing antenatal services as a result of their socio-demographic status [8, 10, 24, 25].

Contrary to much research on the topic [24] our retrospective data could not find a statistically significant relationship between late booking and poor fetal outcomes when other confounding factors linked to poor obstetric outcomes such as ethnicity, maternal age, smoking status etc. were accounted for. Therefore, we propose that late booking may not be the cause of poor outcome, per se, but is in itself a consequence of adverse sociodemographics that, in turn, lead to poor fetal outcomes.

There were limitations in our study. Data for our retrospective study was accessed via Protos database [26] which is completed by the healthcare professional, hence a potential for human error in the data input. Non-availability of medical notes, made it difficult to exclude cases that might have influenced the results. For instance, the retrospective nature of the report has precluded the identification of specific groups of women who are most likely to fail to access antenatal care such as travellers, drug-misusing women, HIV positive women and asylum seekers [27]. Nonetheless, the study involved a large cohort of women ensuring sufficient representation from minority ethnic groups who are more resistant to agreeing to participate in prospective studies as we found out when we prospectively assessed psychosocial status. Thus, the retrospective review enabled a reasonable number of women from non-white backgrounds to be studied.

The prospective arm of the study showed that multiparty and single parenthood, were the strongest predictors of late presentation for antenatal care. Women who booked late demonstrated significantly lower levels of social support compared to early bookers (*P* = 0.047). This observation suggests that lack of sufficient social support resulted in these women prioritising other daily chores over and above accessing antenatal care. This finding is in agreement with our recent qualitative interview study exploring, from the perspective of the pregnant women themselves, why women present late for prenatal care [1]. In that study we reported that women who struggled to cope with difficult personal circumstances, and where support was perceived to be lacking, were reluctant to reveal the pregnancy, and thus to access care, for fear of disapproval, rejection or “consequences”. However, many women volunteered that they simply had other priorities in their lives and had made the decision to avoid or postpone antenatal care. Taken together that study highlighted the need for “coping strategies” as a means of addressing the social support gap that appears to contribute to late pregnancy booking.

Our prospective study had several limitations. The small sample size and some selection bias (more women presenting late, often from ethnic minority groups, declined to participate) may have affected our observations and limited the statistical power to detect some significant associations. For example, the mean score on the anxiety data for both groups was relatively low compared to scores reported previously by other studies [28]. A review conducted for the Royal College of Obstetricians and Gynaecologists [12] had shown anxiety to be one of the barriers to accessing healthcare for recent immigrants in the UK. Because the prospective study involved completion of a questionnaire, as well as obtaining informed consent, many women who could not speak English declined to participate. Future studies will require translator services employing validated survey instruments for use in disadvantaged sociocultural settings. We defined late bookers as women presenting >14 weeks gestation for the prospective study (more in keeping with UK NICE guidance) as opposed to > 20 weeks gestation chosen for the retrospective study (to better demonstrate any demographic and outcome differences between markedly disparate EB and LB groups). This widened the recruitment field for the prospective study but introduced some inconsistency in extrapolating findings between the two studies. Nonetheless, the main strength of our prospective work was compiling scores of many scales to create an overall psychosocial profile of women who booked later for antenatal care, taking into account many confounding reasons for which women may seek care later in pregnancy [29]. Exploring such psychosocial factors and profiles that affect engagement with pregnancy care warrant further study on larger population cohorts.

When the relationship between socio-demographic factors and psychosocial health was explored, our data showed that increasing parity was associated with lower fetal attachment levels for late bookers. This finding is in agreement with Nicols et al. [30] who found that multiparous women had significantly lower fetal attachment scores, possibly due to reduced focus on the current pregnancy in the presence of a young child. Studies have also shown significant differences in social support between ethnicities [31] and marital status [32]. However, possibly due to small sample sizes we did not observe such a relationship.

Previous studies have shown a significant effect of social support on psychological health and levels of depression [29]. In agreement with this literature, our study demonstrated a positive correlation between maternal social support, maternal attachment score, self-esteem and perceived mental health (*P* < 0.001) and a significant negative correlation between social support and anxiety levels (*P* < 0.001) in both groups.

## Conclusions

We have identified that high IMD score is an independent predictor of poor access to antenatal care. Our observation provides further evidence of the existence of inequalities in healthcare availability and access. Further research is required to explore how interventions can improve pregnancy care in socially deprived communities including ethnic minority populations. Such research will need to employ validated survey instruments for non-English speaking antenatal service users and may need to be based in the community to include hard-to-reach families.

We have also highlighted the negative impact of poor social support and social deprivation on maternal sense of wellbeing, esteem and fetal attachment, demonstrating that these sometimes manifest as late presentation for pregnancy care. Our observations highlight the need for larger scale prospective studies exploring the relationship between maternal psychosocial health indices on the one hand and engagement with antenatal care on the other. The initiation of specific services targeted at women and families in communities with high indices of multiple deprivation and unemployment could improve maternal wellbeing and clinical outcomes.

## Additional files

Additional file 1: Appendix 1. Descriptive statistics of Maternal antenatal attachment scale (MAAS) by group. (DOC 62 kb) Additional file 2: Appendix 2. Descriptive statistics of maternal social support scale (MSSS) score by group. (DOC 37 kb) Additional file 3: Appendix 3. Descriptive statistics for Rosenberg self-esteem scale RSES by group. (DOC 45 kb) Additional file 4: Appendix 4. Descriptive statistics of state trait anxiety inventory scale STAI by group. (DOC 61 kb) Additional file 5: Appendix 5. Descriptive statistics of short form health survey SF-12 by group (DOCX 32 kb) (DOC 29 kb)

## Abbreviations

CEMD: The confidential enquiries into maternal deaths
UK: United Kingdom
NICE: National Institute of Clinical Excellence
JW: Jessop wing
EBs: Early bookers
LBs: Late bookers
IMD: Index of multiple deprivation
LSOA: Lower super output area
MAAS: Maternal antenatal attachment scale
MSSS: Maternity social support scale
STAI: State trait anxiety inventory scale
RSES: Rosenberg self-esteem scale
SF-12: Short form health survey
SCBU: Special care baby unit
