# Supplementary material for: Deprivation and poor psychosocial support are key determinants of late antenatal presentation and poor fetal outcomes-a combined retrospective and prospective study
Source: BMC Pregnancy Childbirth. 2015 Nov 25;15:309. doi: 10.1186/s12884-015-0753-3 (PMC4660789; doi:10.1186/s12884-015-0753-3)
Supplement: Additional file 1: Appendix 1. — Descriptive statistics of Maternal antenatal attachment scale (MAAS) by group. (DOC 62 kb) [file 12884_2015_753_MOESM1_ESM.doc]

Additional file 1: Appendix 1: Descriptive statistics of Maternal antenatal attachment scale (MAAS) by group

|  | Gestational Age at Booking | | | | | | | | |
| --- | --- | --- | --- | --- | --- | --- | --- | --- | --- |
|  | Early Booking | | | | Late Booking | | | |  |
|  | Mean | Standard Deviation | Median | Range | Mean | Standard Deviation | Median | Range | Mann Whitney  P value |
| Times that had thoughts about baby | 3.84 | 0.98 | 4 | 1-5 | 3.77 | 0.80 | 4 | 2-5 | 0.443 |
| Feelings when speaking about baby | 3.89 | 0.77 | 4 | 1-5 | 3.50 | 0.90 | 4 | 1-5 | **0.008** |
| Feelings about baby | 4.19 | 0.79 | 4 | 2-5 | 4.25 | 0.78 | 4 | 3-5 | 0.686 |
| Desire to read/get information | 4.09 | 0.88 | 4 | 2-5 | 4.09 | 1.01 | 4 | 1-5 | 0.737 |
| Times picturing baby | 3.12 | 1.08 | 3 | 1-5 | 3.30 | 1.07 | 3 | 5-5 | 0.469 |
| Picturing Baby | 4.05 | 1.14 | 4 | 1-5 | 4.20 | 1.15 | 5 | 2-5 | 0.298 |
| Baby is dependent on me | 4.48 | 0.88 | 5 | 1-5 | 4.57 | 0.82 | 5 | 1-5 | 0.505 |
| Talking to baby when alone | 2.03 | 1.13 | 2 | 1-5 | 1.98 | 1.21 | 2 | 1-5 | 0.590 |
| Thoughts about baby | 4.73 | 0.60 | 5 | 3-5 | 4.61 | 0.89 | 5 | 0-5 | 0.473 |
| Picture of what baby is like in womb | 4.21 | 0.83 | 4 | 1-5 | 4.16 | 0.91 | 4 | 1-5 | 0.861 |
| Feelings of baby inside | 4.54 | 0.72 | 5 | 2-5 | 4.45 | 0.66 | 5 | 3-5 | 0.307 |
| Want to hurt/punish the baby | 4.98 | 0.13 | 5 | 4-5 | 4.95 | 0.30 | 5 | 3-5 | 0.803 |
| Emotionally close/distant | 4.15 | 0.93 | 4 | 1-5 | 4.30 | 0.67 | 4 | 3-5 | 0.596 |
| Good Diet | 4.05 | 0.74 | 4 | 2-5 | 4.14 | 0.60 | 4 | 3-5 | 0.650 |
| After see baby for the first time | 4.88 | 0.32 | 5 | 4-5 | 4.86 | 0.35 | 5 | 4-5 | 0.677 |
| Want to hold baby | 4.73 | 0.60 | 5 | 2-5 | 4.75 | 0.58 | 5 | 3-5 | 0.801 |
| Dreams about pregnancy/baby | 2.47 | 1.26 | 2 | 1-5 | 2.18 | 1.19 | 2 | 1-5 | 0.189 |
| Rubbing stomach where baby is | 3.94 | 1.02 | 4 | 1-5 | 4.07 | 0.90 | 4 | 2-5 | 0.614 |
| If Pregnancy was lost | 4.81 | 0.66 | 5 | 2-5 | 4.89 | 0.32 | 5 | 4-5 | 0.610 |
| MAAS total score | 77.23 | 8.21 | 79 | 52-95 | 76.79 | 7.52 | 76 | 56-90 | 0.699 |
