# Supplementary material for: Deprivation and poor psychosocial support are key determinants of late antenatal presentation and poor fetal outcomes-a combined retrospective and prospective study
Source: BMC Pregnancy Childbirth. 2015 Nov 25;15:309. doi: 10.1186/s12884-015-0753-3 (PMC4660789; doi:10.1186/s12884-015-0753-3)
Supplement: Additional file 2: Appendix 2. — Descriptive statistics of maternal social support scale (MSSS) score by group. (DOC 37 kb) [file 12884_2015_753_MOESM2_ESM.doc]

Additional file 2: Appendix 2: Descriptive statistics of maternal social support scale (MSSS) score by group

| MSSS Questions | Gestational Age at Booking | | | | | | | | |
| --- | --- | --- | --- | --- | --- | --- | --- | --- | --- |
|  | Early Booking | | | | Late Booking | | | | Mann Whitney |
|  | Mean | Standard Deviation | Median | Range | Mean | Standard Deviation | Median | Range | P value |
| Good Friends that support | 4.49 | 0.83 | 5 | 0-5 | 4.43 | 0.85 | 5 | 1-5 | 0.580 |
| Family are always there | 4.75 | 0.60 | 5 | 2-5 | 4.45 | 1.04 | 5 | 1-5 | 0.074 |
| Husband/Partner helps a lot | 4.77 | 0.60 | 5 | 1-5 | 4.43 | 1.35 | 5 | 0-5 | 0.292 |
| Conflict with Husband/Partner | 4.57 | 0.68 | 5 | 2-5 | 4.02 | 1.47 | 5 | 0-5 | **0.042** |
| Feel Controlled by Husband/Partner | 4.82 | 0.57 | 5 | 1-5 | 4.55 | 1.28 | 5 | 0-5 | 0.313 |
| Feel Loved by Husband/Partner | 4.78 | 0.66 | 5 | 0-5 | 4.43 | 1.32 | 5 | 0-5 | 0.088 |
| MSSS total score | 28.18 | 2.56 | 29 | 18-30 | 26.32 | 5.68 | 29 | 6-30 | 0.099 |
