# Supplementary material for: Deprivation and poor psychosocial support are key determinants of late antenatal presentation and poor fetal outcomes-a combined retrospective and prospective study
Source: BMC Pregnancy Childbirth. 2015 Nov 25;15:309. doi: 10.1186/s12884-015-0753-3 (PMC4660789; doi:10.1186/s12884-015-0753-3)
Supplement: Additional file 3: Appendix 3. — Descriptive statistics for Rosenberg self-esteem scale RSES by group. (DOC 45 kb) [file 12884_2015_753_MOESM3_ESM.doc]

Additional file 3: Appendix 3: Descriptive statistics for Rosenberg self-esteem scale RSES by group

|  | Gestational Age at Booking | | | | | | | |  |
| --- | --- | --- | --- | --- | --- | --- | --- | --- | --- |
|  | Early Booking | | | | Late Booking | | | | Mann Whitney |
|  | Mean | Standard Deviation | Median | Range | Mean | Standard Deviation | Median | Range | P value |
| Satisfied with self | 2.32 | 0.57 | 2 | 0-3 | 2.45 | 0.63 | 3 | 1-3 | 0.154 |
| At times think of self as no good | 2.14 | 0.85 | 2 | 0-3 | 2.30 | 0.73 | 2 | 0-3 | 0.356 |
| Number of good qualities | 2.36 | 0.54 | 2 | 1-3 | 2.36 | 0.53 | 2 | 1-3 | 0.995 |
| Able to do things as well as others | 2.39 | 0.60 | 2 | 0-3 | 2.48 | 0.55 | 2 | 1-3 | 0.434 |
| Have not much to be proud of | 2.38 | 0.71 | 2 | 0-3 | 2.57 | 0.62 | 3 | 1-3 | 0.116 |
| Feel useless at times | 2.21 | 0.85 | 2 | 0-3 | 2.34 | 0.81 | 3 | 0-3 | 0.377 |
| A person of worth | 2.46 | 0.58 | 3 | 1-3 | 2.36 | 0.72 | 2 | 0-3 | 0.587 |
| Wish to have more respect for self | 2.17 | 0.74 | 2 | 2-3 | 2.34 | 0.71 | 2 | 0-3 | 0.156 |
| Inclined to feel a failure | 2.58 | 0.64 | 3 | 0-3 | 2.59 | 0.62 | 3 | 0-3 | 0.971 |
| Positive attitude to self | 2.35 | 0.59 | 2 | 1-3 | 2.30 | 0.67 | 2 | 1-3 | 0.713 |
| RSES total score | 20.89 | 4.60 | 21 | 8-27 | 21.64 | 4.47 | 23 | 12-27 | 0.375 |
