# Supplementary material for: Deprivation and poor psychosocial support are key determinants of late antenatal presentation and poor fetal outcomes-a combined retrospective and prospective study
Source: BMC Pregnancy Childbirth. 2015 Nov 25;15:309. doi: 10.1186/s12884-015-0753-3 (PMC4660789; doi:10.1186/s12884-015-0753-3)
Supplement: Additional file 4: Appendix 4. — Descriptive statistics of state trait anxiety inventory scale STAI by group. (DOC 61 kb) [file 12884_2015_753_MOESM4_ESM.doc]

Additional file 4: Appendix 4: Descriptive statistics of state trait anxiety inventory scale STAI by group

|  | Gestational Age at Booking | | | | | | | |  |
| --- | --- | --- | --- | --- | --- | --- | --- | --- | --- |
|  | Early Booking | | | | Late Booking | | | | Mann Whitney |
|  | Mean | Standard Deviation | Median | Range | Mean | Standard Deviation | Median | Range | P value |
| Calm | 1.69 | 0.71 | 2 | 1-3 | 1.61 | 0.75 | 1 | 1-4 | 0.415 |
| Secure | 1.38 | 0.60 | 1 | 1-3 | 1.43 | 0.82 | 1 | 1-4 | 0.799 |
| Tense | 1.82 | 0.80 | 2 | 1-4 | 1.89 | 0.87 | 2 | 1-4 | 0.790 |
| Strained | 1.65 | 0.85 | 1 | 1-4 | 1.39 | 0.72 | 1 | 1-4 | 0.067 |
| At ease | 1.82 | 0.71 | 2 | 1-4 | 1.80 | 0.98 | 1 | 1-4 | 0.384 |
| Upset | 1.35 | 0.74 | 1 | 1-4 | 1.30 | 0.70 | 1 | 1-4 | 0.563 |
| Worrying over misfortunes | 1.93 | 0.92 | 2 | 1-4 | 1.93 | 0.87 | 2 | 1-4 | 0.896 |
| Satisfied | 1.68 | 0.80 | 1 | 1-4 | 1.57 | 0.76 | 1 | 1-4 | 0.416 |
| Frightened | 1.77 | 0.87 | 2 | 1-4 | 1.64 | 0.81 | 1 | 1-4 | 0.389 |
| Uncomfortable | 1.47 | 0.86 | 1 | 1-4 | 1.32 | 0.60 | 1 | 1-3 | 0.499 |
| Self-Confident | 2.16 | 0.92 | 2 | 1-4 | 2.16 | 0.96 | 2 | 1-4 | 0.930 |
| Nervous | 1.99 | 0.80 | 2 | 1-4 | 2.00 | 0.78 | 2 | 1-4 | 0.926 |
| Jittery | 1.55 | 0.74 | 1 | 1-3 | 1.53 | 0.74 | 1 | 1-3 | 0.913 |
| Indecisive | 1.57 | 0.80 | 1 | 1-4 | 1.49 | 0.86 | 1 | 1-4 | 0.383 |
| Relaxed | 1.91 | 0.84 | 2 | 1-4 | 1.89 | 0.92 | 2 | 1-4 | 0.743 |
| Content | 1.64 | 0.81 | 1 | 1-4 | 1.81 | 0.96 | 2 | 1-4 | 0.368 |
| Worried | 1.76 | 0.78 | 2 | 1-4 | 1.70 | 0.70 | 2 | 1-3 | 0.801 |
| Confused | 1.31 | 0.63 | 1 | 1-4 | 1.34 | 0.71 | 1 | 1-4 | 0.949 |
| Steady | 1.93 | 0.78 | 2 | 1-4 | 1.93 | 0.95 | 2 | 1-4 | 0.701 |
| Pleasant | 1.72 | 0.72 | 2 | 1-4 | 1.73 | 0.85 | 2 | 1-4 | 0.786 |
| STAI total score | 34.65 | 10.12 | 32 | 20-66 | 33.51 | 10.26 | 31 | 21-69 | 0.455 |
