# Supplementary material for: Deprivation and poor psychosocial support are key determinants of late antenatal presentation and poor fetal outcomes-a combined retrospective and prospective study
Source: BMC Pregnancy Childbirth. 2015 Nov 25;15:309. doi: 10.1186/s12884-015-0753-3 (PMC4660789; doi:10.1186/s12884-015-0753-3)
Supplement: Additional file 5: Appendix 5. — Descriptive statistics of short form health survey SF-12 by group (DOCX 32 kb) (DOC 29 kb) [file 12884_2015_753_MOESM5_ESM.doc]

Additional file 5: Appendix 5: descriptive statistics of short form health survey SF-12 by group

|  | Gestational Age at Booking | | | | | | | |  |
| --- | --- | --- | --- | --- | --- | --- | --- | --- | --- |
|  | Early Booking | | | | Late Booking | | | | Mann Whitney |
|  | Mean | Standard Deviation | Median | Range | Mean | Standard Deviation | Median | Range | P value |
| UK SF-12 STANDARDIZED PHYSICAL COMPONENT SCALE | 43.96 | 11.16 | 47.17 | 10.97-60.31 | 47.22 | 9.24 | 50.50 | 20.99-58.51 | 0.120 |
| UK SF-12 STANDARDIZED MENTAL COMPONENT SCALE | 49.04 | 10.38 | 49.74 | 18.88-68.05 | 50.62 | 8.87 | 52.41 | 21.28-62.56 | 0.318 |
